# Family Jars

**Published:** 1897-05

**Authors:** 


Family Jars.—It was a sad day for the great and only original Shrady when he was indiscreet enough to chuck a chip at the editor of the *Journal of the American Medical Association*, —"the little man with the big head." Hamilton is nothing, if not versatile. He is well read, and it will not do to tackle him unless one is very sure of his data, which, it seems, Shrady was not. The indiscretion of criticising Hamilton by our distinguished "head of the journalist's guild," Shrady, is only comparable to that of the urchin who thought it would be ever so funny to tickle the mules's heels.

Dr. Shrady made a good deal of fun of something Dr. Hamilton had said, and apparently enjoyed it immensely. He let his gaps down, however, in an editorial on "Osteopathy," which gave Hamilton the opportunity,—which he was not slow to seize,—to show that what Dr. Shrady doesn't know of the history of medicine would make a very large book. In closing his review of Dr. Shrady's editorial on "Osteopathy" (*New York Medical Record*, April 3, 1897), Dr. Hamilton says (*Journal of the American Medical Association*, April 10, 1897):

"The *Medical Record*, however, should not be too severely criticised for its ignorance of 'Osteopathy,' albeit a female 'osteopath,' or 'bone-setter,' in the days of the editor's youth, created a great furore in New York City. The *Medical Record's* knowledge of either medical history or medical science is, it must charitably be admitted, an unstable quantity. It revived Alexander II., after his encounter with the nihilist, by the use of 'oxygen sulphate.' A medical journal capable of such a chemical extravagance can hardly be taken seriously on any subject."

